# A Cognitive Computational Approach to Social and Collective Decision-Making

**DOI:** 10.1177/17456916231186964

**Published:** 2023-09-06

**Authors:** Alan N. Tump, Dominik Deffner, Timothy J. Pleskac, Pawel Romanczuk, Ralf H. J. M. Kurvers

**Affiliations:** 1 Center for Adaptive Rationality, Max Planck Institute for Human Development; 2 Science of Intelligence, Technische Universität Berlin; 3 Department of Psychology, University of Kansas; 4 Institute for Theoretical Biology, Department of Biology, Humboldt Universität zu Berlin; 5 Bernstein Center for Computational Neuroscience Berlin

**Keywords:** cognitive modeling, drift-diffusion model, reinforcement learning, groups, collective dynamics

## Abstract

Collective dynamics play a key role in everyday decision-making. Whether social influence promotes the spread of accurate information and ultimately results in adaptive behavior or leads to false information cascades and maladaptive social contagion strongly depends on the cognitive mechanisms underlying social interactions. Here we argue that cognitive modeling, in tandem with experiments that allow collective dynamics to emerge, can mechanistically link cognitive processes at the individual and collective levels. We illustrate the strength of this cognitive computational approach with two highly successful cognitive models that have been applied to interactive group experiments: evidence-accumulation and reinforcement-learning models. We show how these approaches make it possible to simultaneously study (a) how individual cognition drives social systems, (b) how social systems drive individual cognition, and (c) the dynamic feedback processes between the two layers.

Many decisions are embedded in social contexts, such as sharing news on social media, choosing an investment fund, or deciding whether to jaywalk at a busy intersection in the presence of others (Cialdini & Goldstein, 2004). Individuals are embedded within groups, platforms, and other social entities that they both influence and are influenced by, and these processes of reciprocal social influence shape the dynamics of social systems. Indeed, the collective-intelligence phenomena that emerge from these interactions underpin the immense ecological success—but also destructiveness—of the human species (Henrich & McElreath, 2003; Tomasello et al., 1993). In today’s interactive world, these dynamically evolving social processes seem more important than ever, both online and offline. We argue that to understand collective systems, research needs to adopt approaches that allow complex social dynamics to evolve—that is, to study simultaneously (a) how individual cognition drives social systems, (b) how social systems drive individual cognition, and (c) the interaction between both layers. We argue that to test competing hypotheses in such complex systems, computational approaches are needed that explicitly account for this dynamic feedback across levels.

Though it is widely agreed that the behavior of individuals is the basis of all collective dynamics, many influential models of collective behavior, such as the Vicsek model (Liggett, 1997) or the Voter model (Castellano, Muñoz, et al., 2009), make highly idealized, simplifying assumptions about the underlying individual decision processes. For example, borrowing concepts from statistical physics or epidemiology, these models assume that individuals interact like Brownian particles (Romanczuk et al., 2012) or that information and opinions spread like virulent diseases (Cavalli-Sforza & Feldman, 1981). Such assumptions facilitate mathematical tractability and allow the modeling of collective behavior at large scales. These approaches typically focus on emergent patterns in collectives with very large numbers of individuals. These simulation-driven analyses have provided valuable insights into collective phenomena such as swarms or large herds (Castellano, Fortunato, & Loreto, 2009). However, they typically neglect individual cognition, assuming that large collectives show general properties irrespective of the details of the individual-level decision process. Empirical studies on social cognition have made great progress in understanding the cognitive mechanisms of decision processes in social settings—for example, showing when individuals seek social information (Kendal et al., 2018) and how social influence can alter evaluation processes (Germar et al., 2016) and risk attitudes (Ciranka & Van den Bos, 2019). Although such studies provide detailed insights into how cognition acts in social settings, very few studies take a dynamic approach to study how cognitive processes shape—and are shaped by—collective dynamics over time. Instead, social-cognition studies typically look at static—often simulated—sources of social information and are unable to accommodate the complexity of multiple individuals interacting dynamically and repeatedly in real time. We argue that (a) designing more “dynamic” cognitive-behavioral experiments in which participants repeatedly interact in real time (embracing natural variation in the timing of decisions) and respond to the unfolding social environment created by the decisions of others (as opposed to more “static” experiments that constrain decision timing and prevent recurrent feedback between the choices of group members) and (b) modeling the cognitive processes of such dynamically interacting individuals is a fruitful path forward, making it possible to describe, understand, and predict collective outcomes (Krause et al., 2021).

Computational cognitive modeling has become firmly established as an invaluable tool for studying human decision-making (for introductions to cognitive modeling, see Farrell & Lewandowsky, 2018; Lee & Wagenmakers, 2014; Wilson & Collins, 2019). We argue that taking such models to the level of social interactions will prove instrumental for integrating theories across disciplines such as psychology, biology, and economics within a single framework. Recent advances in cognitive modeling and the increasing availability of software such as Stan (Carpenter et al., 2017), PyMC3 (Salvatier et al., 2016), and Turing (Ge et al., 2018) allow formalizing and testing models to capture these complex interactions (Farrell & Lewandowsky, 2018; Lee & Wagenmakers, 2014) and thus to investigate the bottom-up relationship between individual cognition and collectives: The models take cognitive processes at the individual level as the starting point while simultaneously accounting for the collective dynamics that these processes create. This approach can thus bridge the gap between individual and collective dynamics in social groups. Starting from formal models of individual cognition and mechanistically linking the individual and collective level, this approach makes it possible to analyze dynamic behavior in social experiments and to test quantitative predictions derived from different theories against each other.

We illustrate the strength of this approach with two classes of models that have been highly successful in explaining individual decision-making: evidence-accumulation models that predict trial-level choices and response times and reinforcement-learning (RL) models that predict dynamic learning processes over repeated choices. We highlight novel research directions throughout.

## Modeling Evidence Accumulation in Collectives

### From individual to social-cognitive modeling

**Fig. 1.**
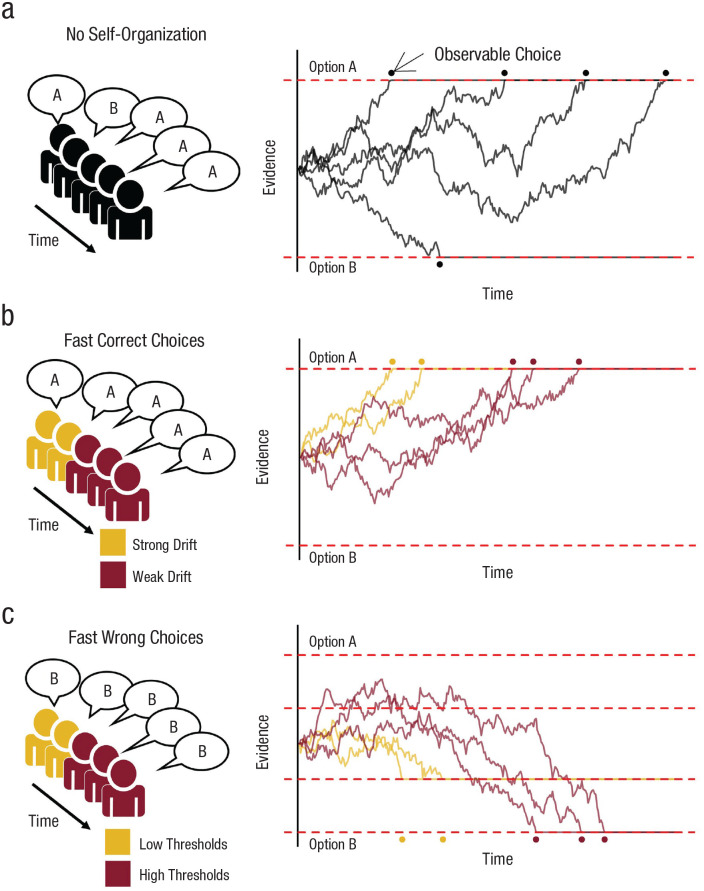
Illustration of the social drift-diffusion model capturing the order of decisions by accounting for the integration of personal and social information during the choice process. Five individuals gather noisy evidence for options A (the correct decision) and B (the incorrect decision). When an individual hits a decision threshold (i.e., red dashed lines), their choice becomes observable (indicated by dots) for the undecided individuals. These undecided individuals can use this information as additional information by drifting to the decision threshold favored by the majority (a). If group members vary in their ability to extract personal information (i.e., their personal drift rate), those with a higher ability are expected to make faster and more accurate choices. This allows groups to self-organize, whereby accurate individuals provide high-quality information to less skilled individuals (b). If group members differ in the amount of information needed to make a decision (i.e., different thresholds), individuals with lower thresholds are expected to make faster but less accurate choices, undermining the accuracy of later-deciding individuals (c). For the code for simulating and plotting, see https://osf.io/xfzqv.

Timing plays a central role in a wide range of decision-making tasks (e.g., animals deciding whether to escape or continue foraging under predation risk or pedestrians deciding whether to wait or cross a busy street). The dominant theoretical framework to account for the underlying individual decision processes is that of evidence-accumulation models. These models describe the decision-making process as a continuous process of accumulating noisy evidence until a decision threshold is reached and a decision is made. The most prominent representative of evidence-accumulation models is the drift-diffusion model (DDM), which captures the choice process between two alternatives (Ratcliff & McKoon, 2008; Ratcliff et al., 2016). It assumes that individuals start with an initial evidence state that can favor one of the decision alternatives (described by the starting point). Over time, they gather further information that changes the evidence state (described by the drift rate) until the evidence for one alternative reaches a level that triggers a decision (by hitting the upper or lower decision threshold; see Fig. 1a). This continuous evidence-accumulation process is typically approximated by assuming small discrete time steps ( Δt ) with the change in the evidence state *L* being described by

(1) $$L\left(t+\Delta t\right)=L\left(t\right)+\delta \times \Delta t+\sqrt{\Delta t}\times \epsilon$$

where δ is the drift rate describing the rate of evidence accumulation per unit of time, with positive (negative) values describing a drift toward the upper (lower) decision threshold. If one option is correct, the drift rate typically describes the rate of evidence accumulation for the correct choice. The parameter *ϵ* adds Gaussian noise and makes the choice process stochastic (i.e., the diffusion process).

By providing an account for response-time distributions and choice probabilities, the DDM is widely used to study individual cognitive processes across many domains (Krajbich & Rangel, 2011; Ratcliff et al., 2016). To date, the large majority of DDM applications have studied single decision makers. Yet many choices are made under social influence, with individuals able to observe the choices of others to inform their own decisions. Animals often observe the flight responses or food choices of conspecifics, and pedestrians observe others dashing across the road. In such situations, early choices can cascade through the group via social contagion (Bikhchandani et al., 1998; Mann, 2018; Poel et al., 2022; Tump et al., 2020). The collective outcome of these dynamics is strongly influenced by how individuals integrate information over time and how they time their decisions. Because DDMs can account for both of these processes, extending such models to the domain of social dynamics is extremely promising.

The integration of personal and social information over time can be described as a dynamic process in which multiple individuals simultaneously collect noisy evidence (see Eq. 1). Once an individual makes a decision, this decision becomes observable for undecided individuals and can be incorporated as further evidence (Fig. 1a). Formally, the incorporation of this additional evidence can be described by a change in the drift rate δ(*t*) as a function of the majority size M(t) of the individuals who already decided at time point *t*:

(2) $$\delta \left(t\right)=\delta_{p}+\delta_{s}\left(M\left(t\right)\right)$$

Thus, the drift rate δ consists of a personal drift rate $\delta_{p}$ and a social drift rate $\delta_{s}$ describing the uptake of nonsocial and social information, respectively. The former describes personal information intake from the task-specific stimuli, whereas the latter describes the social influence that changes with the majority size M(t) :

(3) $$M\left(t\right)=N_{A}\left(t\right)-N_{B}\left(t\right)$$

where $N_{A}(t)$ and $N_{B}(t)$ are the number of individuals who have already decided for the options A or B, respectively. For the simulations in Figure 1 we assumed a linear relationship between the majority size and social-information uptake (i.e., $\delta_{s}=s\times M(t)$ ), with *s* scaling the strength of social-information use. In reality, individuals may use more complex social-information integration strategies, as discussed later.

The social DDM links evidence accumulation at the individual level to the collective level, explicitly modeling the timing of choices and the arrival of new social information, thereby shedding light on the information flow in sequentially deciding collectives (Bidari et al., 2022; Caginalp & Doiron, 2017; Karamched, Stickler, et al., 2020; Karamched, Stolarczyk, et al., 2020; Tump et al., 2020). This framework goes beyond previous models on information cascades, which usually assume a random decision order (Bikhchandani et al., 1998; Deneubourg et al., 1990; Sumpter & Pratt, 2009; but see Vicente-Page et al., 2018). Such models neglect the influence of individual cognitive processes on strategic decision timing. Whether individuals decide early or late can be the result of distinct cognitive processes, such as individual differences in response biases (e.g., starting evidence accumulation closer to one option), personal drift rate, thresholds (also known as speed-accuracy trade-offs), or the integration of social information (Bogacz et al., 2010). Importantly, these cognitive processes are predicted to have different ramifications at the collective level. For example, individual differences in how quickly group members can extract information for the correct option (i.e., variation in expertise) is described by individual differences in the personal drift rate $\delta_{p}$ . Such individual differences in expertise are expected to promote collective intelligence because they will allow “expert” individuals to make early, accurate choices that can then be adopted by later-deciding, less skilled individuals (Fig. 1b). But the social DDM can also predict when social interactions might undermine accuracy. For example, individuals’ preferences for speed or accuracy are reflected in their decision thresholds, with higher thresholds resulting in more accurate, but slower, decisions. If group members vary in these preferences, those individuals with low thresholds are predicted to make early, error-prone choices, thereby jeopardizing the accuracy of individuals with higher thresholds (Fig. 1c). The social DDM allows testing these different predictions by fitting the model to empirical data. It can thereby tease apart distinct decision processes, paving the way for more in-depth modeling and understanding of information cascades or other social processes.

### Strategies underlying social-information integration

How individuals integrate social information crucially determines the outcome of social interactions. Applying a DDM perspective can facilitate a detailed mechanistic understanding and provide novel insights into decision processes in sequentially deciding groups, including (a) social-learning rules, (b) the information present in response order and speed, and (c) information flow.

Social psychology has a long tradition of investigating how the number of individuals displaying a certain behavior influences the likelihood of another individual adopting that behavior (Asch, 1951; Milgram et al., 1969; Morgan & Laland, 2012). This relationship has been speculated to follow different forms, such as a saturating power function (Latané, 1981) or an S-shaped function (Bond, 2005). Yet few studies have allowed social information to arise from the interactions between group members. In more realistic settings, how social information arises will crucially depend on the cognitive strategies of the group members. For example, individuals using quorum thresholds will down-weight small majorities but ramp up copying once the majority reaches a critical size. The choices of these groups will initially be relatively independent, allowing individual errors to cancel out and later-deciding individuals to benefit from often accurate majorities. In contrast, the choices of groups relying on strong copying of small majorities will be highly dependent, increasing decision speed but at the cost of accuracy (Sumpter, 2006; Sumpter & Pratt, 2009). Thus, how much weight individuals give to an observed choice depends on when it was observed and the implemented strategy. In some contexts, choices might be observed before the process of personal evidence accumulation starts. In this case, the social evidence can enter the choice process by biasing the starting point instead of the drift that can push people toward confirming the social source without thoroughly evaluating their personal information (Germar & Mojzisch, 2019). By operationalizing these strategies with the social DDM, future research can measure and test the use of such strategies. For example, the diminishing effect of each additionally observed choice described by Latané (1981) via a saturating power function—but also other relationships—can be implemented via the social drift rate (see also Tump et al., 2020):

(4) $$\delta_{s}=s\times M{\left(t\right)}^{q}$$

where *s* scales the strength of social influence and *q* influences the shape of the power function.

Freely timed decisions allow individuals to make inferences from response times. For example, the speed of an observed decision is used to infer the decision quality (Frydman & Krajbich, 2022). Similarly, the order in which people make decisions can convey information. For example, a choice that diverges from the current majority is predicted to be based on strong personal information (Mann, 2018). Even the absence of decisions can convey information. When group members start with a bias toward one option, a long period without a choice may indicate that individuals have gathered good evidence for the initially less preferred option (Karamched, Stolarczyk, et al., 2020). Allowing participants to freely time their decision can thus provide a richer and more realistic understanding of social systems.

Taking such a dynamic approach can cast light on the mechanisms driving information flow. Previous work has shown that relying on social information in sequentially deciding groups can increase accuracy (Goeree et al., 2007; Mann, 2021; Tump et al., 2020; Vicente-Page et al., 2018) but that it can also promote false information cascades or maladaptive herding (Anderson & Holt, 1997; Baddeley, 2010; Bikhchandani et al., 1998; Toyokawa et al., 2019; Weizsäcker, 2010). The DDM has the potential to bring together such results under one framework. One driving factor here is the order in which individuals decide, whereby the benefits of social interactions emerge when individuals coordinate their response time according to information quality (i.e., deciding early/late when possessing strong/weak evidence) but fail to emerge when they do not (Gul & Lundholm, 1995; Kurvers et al., 2015; Tump et al., 2020; Vicente-Page et al., 2018; Zhang, 1997).

### The emergence of biases in social systems

The DDM framework has been used extensively to understand the emergence of biases at the individual level (Gold & Shadlen, 2007; Leite & Ratcliff, 2011; Mulder et al., 2012), but few studies have looked at biases in social systems. To illustrate, consider a police officer approaching a potentially dangerous situation and needing to decide whether or not to shoot. Previous research has studied which aspects of the cognitive process can explain biases in single police officers, showing that the starting point and evaluation of incoming information depend on the suspect’s race (Johnson et al., 2018; Pleskac et al., 2018). In reality, however, the decision to shoot is rarely made in isolation. Most police precincts in the United States dispatch at least four officers when a suspect is expected to be armed. Despite the importance of the social context, it remains unknown how biases play out when two or more police officers approach a potentially dangerous scene. Creating realistic social scenarios (e.g., interactive shooting simulations) that acknowledge the role of timing is key for understanding these issues.

The DDM framework can also be used to generate predictions for social contexts based on modeling the behavior of single individuals. For example, experimental research on individual police officers in shooting simulators has shown that the decision to shoot is typically made faster than the decision not to shoot, explained by a bias in the starting point (Pleskac et al., 2018). Such asymmetries in decision timing—which appear in various social contexts (Tump, Pleskac, et al., 2022)—are predicted to have consequences on which decisions (and potential biases) are amplified in a social context because early-arriving social information typically exerts a stronger influence on the collective (Tump, Pleskac, et al., 2022; Tump, Wolf, et al., 2022). The prediction at the collective level is thus that having multiple police officers in a shooting simulator will increase the likelihood to shoot.

### Individual heterogeneity, social networks, and norms

It is well known that individuals differ systematically in many key aspects of cognition (Kanai & Rees, 2011), including how they react to standardized social information (Molleman et al., 2019, 2020). However, few studies have quantified such individual differences in dynamic social systems, let alone studied their importance at the collective level. Cognitive models accounting for social interactions—such as the social DDM—can systematically quantify individual differences and their importance for social systems.

Another key dimension on which individuals may vary is the number and structure of social contacts. The topology of social networks is known to strongly influence the dynamics of information flow (Galesic et al., 2023). By allowing freely timed decisions, future research could address questions such as how the speed of a decision interacts with a network structure and which structures promote (or prevent) the influence of early-deciding individuals (Gross & Blasius, 2008).

Last, the behavior of others can also convey normative expectations. How such expectations are incorporated into the choice process and influence early choices and thereby the social dynamics are largely open questions. For example, individual tendencies to behave selfishly or cooperate have been described to influence the starting point (Chen & Krajbich, 2018), whereas social expectations can bias the drift rate (Germar & Mojzisch, 2019; Toelch et al., 2018), with different expected consequences for emerging dynamics. Because normative influence plays a central role in many everyday social interactions, research is needed to investigate how the cognitive underpinnings promote or discourage undesired social dynamics such as jaywalking, collective violence, and hate speech (Krause et al., 2021).

Although the DDM models two-alternative forced-choice tasks, the approach could be extended to other tasks. For example, models accounting for go/no-go tasks provide an interesting extension (Ratcliff et al., 2018) because only the “go” action is observable by others, which causes an asymmetry in information flow in collectives. Evidence-accumulation models have also been extended to tasks with more than two alternatives (Krajbich & Rangel, 2011; Kvam, 2019). Other extensions can account for different types of social-information exchange—for example, directly communicating evidence states (Bidari et al., 2022). Jointly, these models offer the potential to develop tools that shed new light on temporal coordination and information flow in a much broader range of social systems by explicitly modeling the process of personal and social-information accumulation.

## Modeling Reinforcement Learning in Collectives

### Individual and social learning

**Fig. 2.**
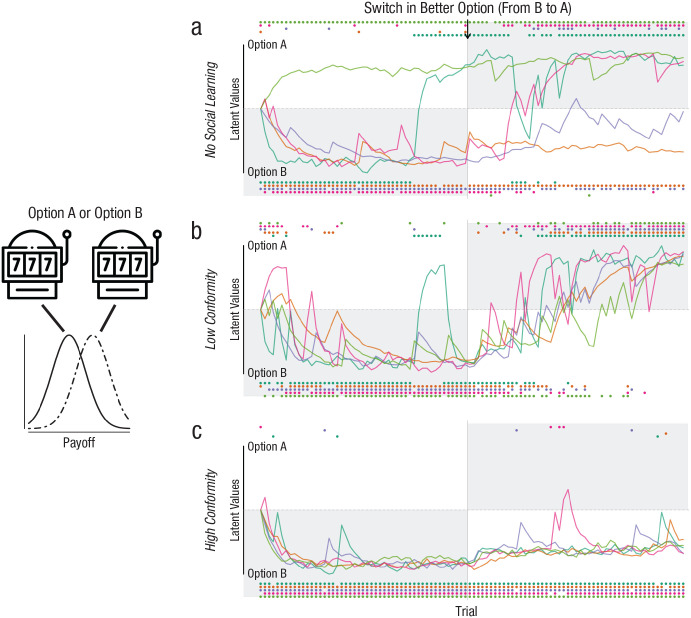
Simulations showing the influence of individual strategies on collective performance in a social reinforcement-learning (RL) model. Five individuals repeatedly choose between options A and B, which differ in their average payoffs ( $\bar{A}=15$ and $\bar{B}=20$ points, with $SD_{AB}=3$ ; see illustration on the left). Following their choices (colored dots), individuals receive payoffs and update their latent values (i.e., beliefs about the value of both options; colored lines; see Eq. 5). After half of the trials, the relative payoffs change, and option A produces, on average, a higher payoff. In addition to this personal information, individuals can observe the choices of other group members and integrate these social cues depending on their learning strategy (see Eqs. 7 and 8). Without social learning (social-learning weight σ=0 ), only some individuals learn which option produces the highest payoff (a; shaded areas). With low-conformist social learning ( σ=0.3 , θ=1.5 ), all individuals initially learn that option B produces a higher payoff and are able to successfully track the change in payoffs (b). With high-conformist social learning ( σ=0.7 , θ=5 ), individuals quickly converge on the initially better option but continue to choose this option after the change in payoffs, illustrating the danger of maladaptive herding in social systems (c). Individual RL parameters were fixed with the updating-rate parameter α=0.3 and exploration/exploitation-rate parameter β=0.18 (see Eqs. 5 and 6). The full simulation and plotting code is available at https://osf.io/xfzqv.

Collective dynamics are not limited to single choices (as typically assumed in evidence-accumulation models). They often unfold throughout many successive choices. To succeed in complex and heterogeneous environments, organisms must continually learn from the consequences of their actions and adjust their behaviors accordingly. Social organisms can learn not only from direct interaction with the environment but also from the observed choices and behaviors of others. Such social learning allows groups to pool information and to adapt to changing conditions more quickly and reliably than individual learners (cf. Figs. 2a and 2b; Boyd et al., 2011; Kendal et al., 2018). However, similar to information cascades in the social DDM, excessive social learning can also result in maladaptive herding, in which collectives continuously copy each other and fail to track the state of the environment (see Fig. 2c; Aoki & Feldman, 2014; Kendal et al., 2018; Rogers, 1988).

To standardize the social information available to participants, many experimental studies investigate social learning in relatively simplistic settings in which social information either comes from earlier participants or is not truly “social” but created by the experimenter (Hoppitt & Laland, 2013; Mesoudi, 2021). This approach has provided important insights into the mechanisms and strategies individuals use to learn from others (for review, see Kendal et al., 2018). However, it does not allow the dynamic collective consequences of individual learning strategies to be studied. To uncover how individuals use social information and how different social-learning strategies shape collective performance, researchers need to study the use of social information in social environments created by individuals, that is, in scenarios in which social information arises endogenously from the behavior of others in interacting groups.

### Dynamic inference for dynamic collectives

RL provides a powerful computational framework that links observed choices to latent individual-level value updating and translates the influence of such latent values (i.e., estimated payoffs for each choice option) into individual-choice probabilities (for a general introduction, see Sutton & Barto, 2018). Standard individual-level RL models consist of two basic components. First, an updating equation describes how latent values (“*Q* values”) of different behavioral options *i* change over time as a function of reward-prediction errors (i.e., differences between received rewards $\pi_{i,t-1}$ and reward predictions):

(5) $$Q_{i,t}=Q_{i,t-1}+\alpha \left(\pi_{i,t-1}-Q_{i,t-1}\right)$$

The updating-rate parameter α determines the relative weight of recent experiences and thus influences how quickly individuals update their values on the basis of recent experiences (colored lines in Fig. 2 illustrate latent value updating). Second, a *softmax* choice rule translates the latent values of different options (here shown for two options) into probabilities that those options are chosen:

(6) $$P_{i,t}=\frac{exp(\beta Q_{i,t})}{{\sum \;}_{m=1}^{2}exp(\beta Q_{m,t})}$$

The exploration/exploitation-rate parameter β describes how strongly differences in latent values determine choices and thus influences an individual’s tendency to select new options over relying on previously rewarding behaviors.

### Linking individual-level learning to group-level adaptation

Extending RL models to dynamic social situations makes it possible to identify mechanistic links between individual-level (nonsocial and social) learning, the social information that such learning creates, and the resulting group-level consequences (e.g., Deffner et al., 2020; McElreath et al., 2005, 2008; Toyokawa et al., 2019; Toyokawa & Gaissmaier, 2022). Social RL models allow different observable social cues to also influence the probability that a certain option is chosen:

(7) $$P_{i,t}=(1-\sigma )\frac{exp(\beta Q_{i,t})}{{\sum \;}_{m=1}^{2}exp(\beta Q_{m,t})}+\sigma P_{S,i,t}$$

An additional parameter σ represents the relative weight individuals place on social information as opposed to their own experience, and the social-choice probability $P_{S,i,t}$ can include the influence of several option- or model-specific social cues such as frequency-dependent (or “conformist”) copying (Hoppitt & Laland, 2013; McElreath et al., 2005):

(8) $$P_{S,i,t}=\frac{n_{i,t-1}^{\theta }}{{\sum \;}_{m=1}^{2}n_{m,t-1}^{\theta }}$$

where $n_{i,t-i}$ is the number of group members previously choosing option *i* and θ is the conformity exponent ( θ>1 represents conformity and 0<θ<1 represents anticonformity). Figure 2 illustrates the influence of different social-learning weights and conformity exponents (for simulation and plotting code, see https://osf.io/xfzqv).

Explicitly modeling the learning and decision processes of individuals in collectives can provide mechanistic insights into collective adaptation and decision-making. For example, it has long been unclear why groups sometimes exhibit collective intelligence and other times maladaptive herding. Toyokawa et al. (2019) studied interactive group experiments in which individuals needed to learn which of three slot machines produced the highest payoff. Fitting social RL models, they showed that the individual-level social-learning strategies explained when groups succeeded (or failed) in adapting to a changing environment (see also Fig. 2). Specifically, a weak conformist bias allowed groups to perform well in stable environments while retaining flexibility when environmental conditions changed. In harder tasks, in which the payoff distributions showed more overlap, individuals showed a stronger conformist bias and thus tended to stick with an outdated maladaptive solution when conditions changed. Further increasing the realism (and complexity) of such experiments, Deffner et al. (2020) allowed for dynamically changing group compositions through movement between groups. Using time-varying social RL models, they showed that individuals relied heavily on conformist social learning after entering a new group with experienced group members but more on personal information when group members did not provide useful social information. In simplifying group dynamics, previous studies may therefore have underestimated the amount of social learning occurring in more realistic, dynamic groups with different experience levels.

Computational models can also help to better understand seemingly paradoxical effects of individual strategies. For example, many RL studies have found that single individuals’ value updating tends to focus on recent experiences, which can bias them to choose safe options that are suboptimal in the long run. Because conformity tends to amplify individual biases, one might expect collectives to display even stronger biases toward safe, suboptimal behavior. If conformist social influence was strong, Toyokawa and Gaissmaier (2022) indeed found that individual risk aversion was amplified in interactive groups. However, if conformist influence was weaker than personal risk aversion, social learning surprisingly increased exploration and promoted favorable risk taking. Only by studying how the learning and choice biases of multiple individuals unfold in dynamically interacting collectives can we understand when collectives mitigate, rather than amplify, individually biased decision-making.

There is a rich diversity of social-learning strategies beyond conformity, such as prestige-based, success-based, similarity-based, and novelty-based learning (Kendal et al., 2018) that can equally be integrated into social RL models via Equation 7. The collective consequences of such strategies are still largely unexplored. For instance, preferentially learning from high-prestige individuals might prove adaptive for the collective when prestige accurately reflects past performance and generalizes to novel tasks but maladaptive in hierarchical groups in which this is not the case. In addition, social-learning strategies do not operate in isolation but jointly influence individuals’ choices. Future research could investigate how different social-learning strategies in concert allow collectives to adapt to changing environments (or prevent them from doing so). Last, group dynamics and social-network structures have proven to be important drivers of social learning and collective adaptation (Chimento et al., 2021; Deffner & McElreath, 2022; Derex & Mesoudi, 2020; Galesic et al., 2023). Experiments on social RL have recently started to move beyond closed groups, but future studies could allow even more complex turnover and network dynamics to emerge, making it possible to investigate how such effects might interact with individual cognition.

### Cognitive details matter: decision biasing, value shaping, and meta-learning

Recent work in cognitive neuroscience has generated novel insights into the mechanisms underpinning people’s abilities to learn from others (for a review, see Olsson et al., 2020). This line of research has suggested how cognitive details might matter for collective outcomes. For example, in all social RL models that have been applied to interactive group experiments (see also Eq. 7), social information directly influences the probability that a certain option is chosen without changing the latent values (e.g., Deffner et al., 2020; McElreath et al., 2005; Toyokawa et al., 2019; Toyokawa & Gaissmaier, 2022). As an alternative to such “decision-biasing” social learning, Najar et al. (2020) proposed a “value-shaping” process in which social information directly enters the latent-value estimation of different options such that observed choice options become inherently more “valuable” to participants (implemented through an extension of Eq. 5) rather than only affecting their behavior. The authors presented experiments that suggested that value shaping might explain human choice behavior in their task better than decision biasing or other social-learning models. More empirical research is needed on the relative importance of decision biasing and value shaping and on their collective-level consequences, which can be tested through evolutionary simulations and dynamic group experiments. Value shaping might lead to faster learning in stable environments by aligning the internal expectations of individuals. In stochastic environments, however, decision biasing might be more adaptive because individuals influence each other’s choices but still update their beliefs about the world on the basis of their personal experience.

Last, going beyond social RL, Wu et al. (2022) argued that the power of human social learning is not underpinned by a single cognitive capacity but by the ability to flexibly switch between different computational modes. Specifically, social learners can directly copy other individuals’ actions without any causal understanding (“policy imitation”), infer the values guiding those actions (“value inference”), or infer their model of the environment and intrinsic rewards (“belief inference and reward inference”). Similarly, human individual learning extends beyond pure Rescorla-Wagner-style updating (Sutton & Barto, 2018), and future work could implement more realistic learning models from computational neuroscience—for instance, models based on variational inference (Blei et al., 2017; Doya et al., 2006). To our knowledge, no research has yet addressed such complex forms of individual and social learning in dynamic interactive experiments. How do collectives adapt to environmental changes if individuals use social information to infer others’ mental states instead of blindly copying their behavior? How do collective outcomes change if people have access to other people’s goals instead of only their actions? And how do social learners decide to switch between different strategies when faced with multiple sources of social information? These and many related questions await more detailed investigation—there is thus vast unexplored territory concerning the collective consequences of the various cognitive (social)-learning mechanisms that facilitate collective adaptation and cultural evolution (Galesic et al., 2023).

### Combining evidence accumulation and reinforcement-learning models

Social-evidence accumulation and social RL models have thus far been used independently only within largely distinct research traditions, although they are in fact highly complementary: RL models describe the dynamics of latent value updating while assuming a simplified decision process (usually a softmax choice rule) that leaves out the complexities of noisy evidence accumulation. In contrast, evidence-accumulation models account for choice probabilities and response times but do not model information transfer about option quality across trials. Combining both models would constitute a major advance for the computational modeling of social systems. Similar RL diffusion models have already been developed for the nonsocial domain, explaining choice behavior better than previous models (Fontanesi et al., 2019; Konovalov & Krajbich, 2016; Pedersen et al., 2017). For example, Pedersen et al. (2017) let the drift rate change depending on (learned) expected rewards through an RL process. They also tested which other features of the DDM process (e.g., the starting point or decision threshold) change with experience by comparing different model versions using cross-validation (for further variations, see Fontanesi et al., 2019). Extending such models to dynamic social systems might provide fresh insights into collective decision-making across different time scales. How does the timing of individual decisions influence the exploration of different options, and how do experienced payoffs and observed choices in turn influence the subsequent evidence accumulation and temporal self-organization of collectives? Integrating these approaches provides exciting prospects for theory development and empirical breakthroughs but also requires researchers to develop and test additional assumptions about the exact intersection of both models and how social environments might influence learning dynamics and choice processes in different ways.

## Conclusion

Human collectives are complex, dynamic systems that emerge from and shape individual cognition. Because of this inherent complexity, most previous studies have either abstracted away from individual cognition and focused on macro-level dynamics or used constrained, simplified experiments to investigate specific aspects of social interactions. Both approaches have been (and will continue to be) fruitful, but focusing exclusively on one side of the dynamic interplay between individuals and collectives will necessarily miss crucial aspects of social dynamics. We have argued that more naturalistic experiments that allow complex social dynamics to unfold over time can close the gap between simulation-based studies on large-scale collective phenomena and experimental studies on individual-level social cognition. More dynamic group experiments will be useful only if appropriate statistical tools are available for their analysis, and complexity of experimental setups should not be an end in itself. Therefore, we advocate for a close integration between experimental designs and computational models.

There are different ways to link computational models to empirical evidence: Most commonly, researchers fit models to data from experiments that closely mirror the structure of the theoretical model to empirically identify plausible parameter values. This approach can measure features of latent cognitive processes such as how fast participants update their beliefs or how sensitive they are to social information; it can also be used to test specific hypotheses if they can be expressed in terms of certain parameter values (e.g., conformity vs. anticonformity depending on the value of θ in Eq. 8). A complementary aim is to test and compare competing model assumptions to assess which assumed processes are compatible with empirical evidence using, for example, model comparison or model selection (Farrell & Lewandowsky, 2018; McElreath, 2020).

The application of computational models to social systems is greatly facilitated when researchers can build on well-grounded models of how (nonsocial) environmental features and individual characteristics influence the decision process, which can then be extended to social contexts to allow for dynamic feedback between individuals’ choices. Most of these established formal models—including all examples discussed in this article—describe decision problems with a well-defined set of potential actions that can be described by probability distributions and, therefore, fitted to experimental data using routine statistical methods. If behavior is even more unconstrained (e.g., free movement across space, naturalistic social interactions or unstructured discussions), single actions can be difficult to pin down, and the direct application of established decision-making models becomes more difficult and potentially intractable. Even if such experiments may not be used to directly test or falsify hypotheses generated by computational models, social-cognitive models can serve as starting guides for exploration and help to identify essential features of complex real-world interactions, limit the problem space, and inspire new experimental paradigms and computational methods.

Last, applying a cognitive computational approach to collective decision-making will not only generate novel advances in basic research but also might help researchers and policymakers to better generalize insights from cognitive science to other social systems and to predict and guide how different collective phenomena unfold in the real world (Deffner et al., 2022; Glymour et al., 2016). From the emergence of economic bubbles to the spread of false news and polarization of beliefs, collective dynamics can go very wrong, leading to herding and false information cascades with devastating societal consequences. Whether interventions successfully reduce the risk of undesired dynamics ultimately depends on features of the decision-making process. On a more positive note, culture and other social dynamics can facilitate flexible adaptation to vastly different environments and (at least partly) underlie our success as a species (Henrich, 2017). Better understanding the circumstances under which collectively intelligent behavior emerges from individual-level cognition will thus be crucial in helping people to adapt and thrive in a changing world.

## Transparency

*Action Editor:* David Garcia

*Editor:* Interim Editorial Panel

*Author Contributions*

A. N. Tump and D. Deffner contributed equally to this manuscript. All of the authors approved the final manuscript for submission.
